# CITED1 expression in odontogenic cysts

**DOI:** 10.1186/s12903-024-04413-4

**Published:** 2024-07-12

**Authors:** Fırat Aşır, Zeki Özalp, Özden Uçtu Yülek, Fikri Erdemci, Tugcan Korak, Fatih Taş

**Affiliations:** 1 Department of Histology and Embryology, Medical Faculty, Dicle University, Diyarbakır, Turkey; 2 Department of Medical Biology, Medical Faculty, Kocaeli University, Kocaeli, Turkey; 3 Department of Histology and Embryology, Medical Faculty, Siirt University, Siirt, Turkey; 4 Department of Pathology, Çanakkale 18 Mart University, Çanakkale, Turkey; 5 Department of Oral, Dental and Maxillofacial Surgery, Siirt Oral and Dental Health Center, Siirt, Turkey

**Keywords:** Odontogenic cyst, Keratocyst, Radicular cyst, Dentigerous cyst, CITED1

## Abstract

**Background:**

Originating from odontogenic tissue, Odontogenic cysts are pathological cavities lined with epithelial cells and surrounded by fibrous connective tissue. This study investigated expression of CITED1 protein in different types of odontogenic cysts.

**Material and method:**

40 keratocysts, 40 radicular cysts, and 40 dentigerous cysts were excised and processed for routine paraffin wax embedding protocol. Macroscopic and panoramic radiographies images were used for diagnosis. Demographical properties and dental parameters were recorded. Cystic tissues were stained with hematoxylin-eosin dye and CITED1 antibody. Semi-quantitative analysis was performed for immune staining. The protein-protein interaction network, hub gene detection and KEGG analysis were conducted using Cytoscape software.

**Result:**

Odontogenic keratocysts was imaged with 6–8 layered epithelial cells and fibrous cyst walls with inflammatory cells. Radicular cysts had stratified squamous epithelium with varying thickness, ciliated cells, and Rushton hyaline bodies. Dentigerous cysts presented hyperplastic non-keratinized epithelium, fibrous tissue, rete ridges, and inflammatory cells. CITED1 immunoexpression was highest in odontogenic keratocysts, followed by radicular cysts, and lowest in dentigerous cysts. Nuclear and cytoplasmic CITED1 expression was significantly elevated in odontogenic keratocysts compared to radicular and dentigerous cysts. The top five targets of CITED1 were identified, primarily showing enrichment in hormone and cancer related pathways.

**Conclusions:**

Positive CITED1 expression in all three types of odontogenic cysts suggest a potential role for CITED1 in the pathogenesis of odontogenic cysts, particularly in keratocysts. Further investigations are needed to elucidate the exact mechanisms underlying the differential expression of CITED1 and its implications for the development and progression of odontogenic cysts.

## Introduction

Odontogenic cysts are common in jaw due to epithelial remnants during odontogenesis [1, 2]. Although their pathogenesis differs for each odontogenic cyst, they are commonly categorized as developmental (such as dentigerous, odontogenic keratocyst) and inflammatory (such as radicular) [3, 4]. Determining frequency of odontogenic cyst in society is challenging. However, a retrospective analysis in United Kingdom (UK) by oral and maxillofacial department showed that their frequency is 13.8% in adults and 11.78% in children [5, 6]. Numerous patients with odontogenic cysts are admitted to departments of oral and maxillofacial pathology, yet they exhibit significant diversity in presentation, size and histological characteristics [7, 8].

Diagnosis of odontogenic cysts are typically histological tissue preparation with macroscopic dissection to enable the best evaluation of the lining epithelium in relation to the underlying cyst wall. A detailed written description of the radiological characteristics is accompanied for an accurate histological diagnosis, encompassing details such as the site, size, outline, locularity, radiolucency/radiopacity, and the relationship with nearby teeth and structures [5, 9].

cAMP-responsive element-binding protein (CBP)/p300-interacting transactivator with glutamic acid (E) and aspartic acid (D)-rich C-terminal domain (CITED) protein family have been shown to play in variety of cellular and developmental processes and responses to environmental stimuli at the transcriptional level in mammals [10]. CITED1, One of the CITED protein family, is a 27-kDa transcriptional co-factor that is specifically expressed in embryonic and extraembryonic tissues during embryogenesis [11, 12]. It doesn’t directly bind DNA however it can interact with DNA-binding proteins [13]. Dunwoodie et al. [14] studied role of CITED1 in mouse embryogenesis and found its expression in progenitors of heart, limb, axial skeleton, and placenta. CITED1 expression was also shown in adult heart and mammary gland, melanocytes, melanoma cells, and papillary thyroid carcinoma [12]. Sun et al. [15] investigated the expression of CITED1 and recorded that CITED1 was associated with hypoxic conditions in embryos, which may potentially lead to inflammation. Some developmental cysts are highly recurrent (odontogenic keratocyst and glandular odontogenic cyst) while other developmental cysts have low recurrence (orthokeratinized odontogenic cyst and lateral periodontal cyst). Their diagnosis is mainly based on radiographic/imaging finding with confirmation of histopathological findings, yet some cystic lesions of the jaws can be detected during clinical examination (bone cortical expansion, tooth mobility, tooth displacement, pain due to infection, etc.) [16–18]. A molecular marker by immunohistochemical technique may help the diagnosis in this manner. Considering their developmental and inflammatory origin, this study aimed to investigate the potential association between the expression of CITED1 in odontogenic cysts with different origins.

## Materials and methods

### Patient selection and collection of cysts

35 keratocysts, 35 radicular cysts, and 35 dentigerous cysts were excised from patients who admitted to Oral and Dental Health Center, Siirt, Turkey. Patients was diagnosed based on clinical and radiographical findings. All patients were over 16 years old. All patients were informed consent to participate in the study. Non-odontogenic lesions were excluded (non-epithelial cysts or tumor). Patients with incomplete medical records, previous treatment (surgical excision, radiotherapy), pregnancy and other systemic infections were also excluded. Samples with moderate to severe chronic inflammatory infiltrate and with a clinical history of infection was excluded in this study. Histological diagnosis of the lesions (odontogenic keratocysts, dentigerous cysts and radicular cysts) was based on the WHO Classification of Head and Neck Tumours (2017).

### Tissue preparation

Tissue preparation process was performed at Department of Histology and Embryology, Dicle University. Odontogenic cysts were fixed in %10 formaldehyde solution and furtherly processed for paraffin embedding tissue protocol. Samples were deparaffinized and passed through descending alcohol grading series and washed in distilled water. Hematoxylin eosin staining were applied to the sections. Sections were quickly immersed in increasing alcohol series (through 80%, 90%, 96%, %100) and cleared in xylene for 3 × 15 min. Sections were mounted and imaged with Zeiss Imager A2 light microscope.

### CITED1 immunostaining

Samples were deparaffinized, dehydrated in descending alcohol series and washed in distilled water. Hydrogen peroxide solution (cat# TA-015-HP, ThermoFischer, US) was dropped onto the sections and incubated for 20 min. Sections were kept in Ultra V Block (cat#TA-015-UB, ThermoFischer, US) solution for 7 min. Sections were washed with primary antibody CITED1 (cat# PA5-65541, ThermoFisher, Fremont, CA, US, dilution ratio:1/100) overnight at + 4 °C. Sections were incubated with biotinylated secondary antibody (cat#TP-015-BN, ThermoFischer, US) for 14 min. Streptavidin-peroxidase (cat#TS-015-HR, ThermoFischer, US) was reacted with sections for 15 min. Diaminobenzidine (DAB) (cat#TA-001-HCX, ThermoFischer, US) was used for chromogen to observe protein expression. Sections were washed with PBS and counterstained with Harris hematoxylin. Slides were mounted and analyzed by Zeiss Imager A2 light microscope. A histological scoring was developed for immunohistochemical staining using H-scoring [19]. 10 areas were selected from each of the samples and analyzed for CITED1 expression. Two blinded histologists were recorded scores, distinctly (0: no expression; 1: mild expression; 2: moderate expression; 3 high expression). Negative control staining was performed as same steps but primary antibody incubation was omitted. Cohen’s Kappa coefficient performed for the calibration of histopathological diagnoses by pathologists (Cohen’s kappa: 0.88).

### Semi-quantitative histological score

The staining intensity (expression) of primary antibodies was measured by Image J software (version 1.53, http://imagej.nih.gov/ij). Signal intensity was measured by the method of Crowe et al. [20]. Quantification was recorded by analyzing 5 fields from each specimen per group according to method described by Aşır et al. [21]. In specimens, the brown color represented positive expression of the antibody of interest while the blue color represented a negative expression of the antibody of interest. Signal intensity from the analyzed field was calculated by dividing the intensity of the antibody of interest by the whole area of the specimen. A value for staining area/whole area was calculated for each specimen from five fields. An average value was measured for each group and recorded as semi-quantitative immunohistochemistry scoring. Slides were imaged with Zeiss Imager A2 light microscope. All images were processed and quantified using ImageJ software.

### Hub gene detection and KEGG pathway enrichment

We constructed a protein-protein interaction (PPI) network of CITED1 and identified its central interactors using Cytoscape v3.10.1 (San Diego, CA, USA). The CITED1 PPI network was generated with a maximum of 50 interactors and a confidence level set at 0.4, indicating medium confidence [22]. The top ten key interactors of CITED1 were analyzed through the CytoHubba plugin using the Maximal Clique Centrality (MCC) algorithm, which has a high performance in precisely estimating core proteins within the PPI network [23]. Using Cytoscape software, we conducted Kyoto Encyclopedia of Genes and Genomes (KEGG) pathway analysis to predict pathways in which central interactors of CITED1 are enriched. Pathways with *p*-values less than 0.05 were considered statistically significantly annotated.

### Statistical analysis

IBM SPSS Statistics version 25.0 software was used for statistical analysis. Data distribution was tested with Shapiro-Wilk test and data was non-normally distributed. Comparisons between groups were tested with Kruskal Wallis and post hoc Dunn’s test. Data were recorded as median (min-max). Significance level was shown as *P ≤ 0.05 and ****, *P* ≤ 0.0001.

## Results

### Features of patients

Some demographical and dental parameters were shown in Table 1. The mean age of patients with odontogenic keratocyst among groups were the youngest. More male patients were enrolled in this study. Most of the odontogenic cysts surgically removed from posterior of mandible of patients. Cortical expansion and cortical destruction were also common findings in patients.

**Table 1:** Demographical properties of patients and parameters of dental cysts

Parameters			
Age, mean (min-max)	Keratocyst		28.50 (22–75)
	Radicular cyst		36.01 (13–49)
	Dentigerous cyst		34.44 (17–72)
Gender, *n* (%)	Male		83 (%69.17)
	Female		37 (%30.83)
Jaw location, *n* (%)	Maxillary	Anterior	9 (%7.50)
		Posterior	15 (%12.50)
	Mandible	Anterior	11 (%9.17)
		Posterior	85 (%70.83)
Cortical expansion, *n* (%)	Yes		48 (%40.00)
	No		72 (%60.00)
Cortical destruction, *n* (%)	Yes		101 (84.17)
	No		19 (15.83)
Surgery	Marsupialization		56 (45.72)
	Enucleation		64 (54.28)

#### Macroscopical and radiological images

**Fig. 1:**
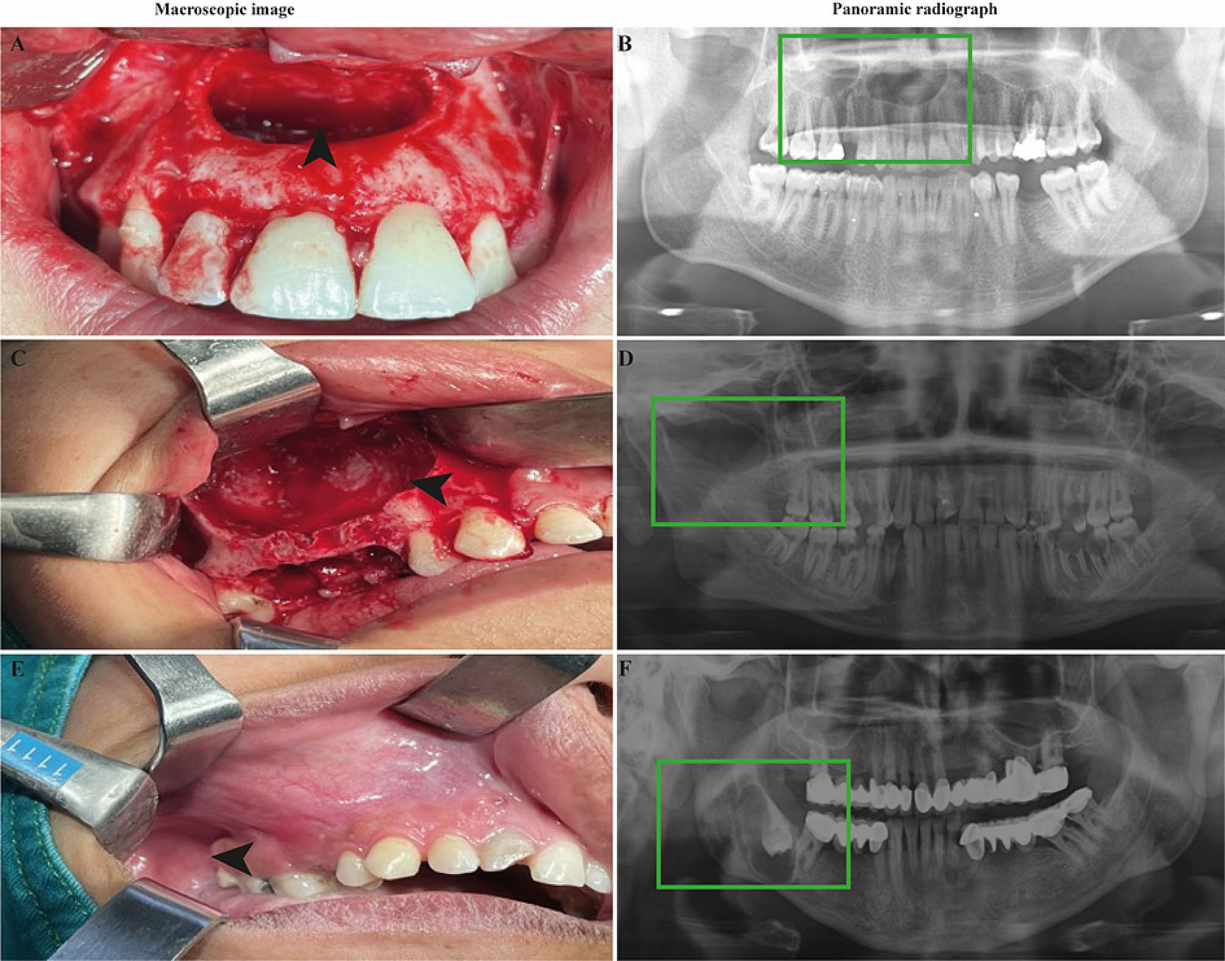
Macroscopic and panoramic radiographical images of patients with odontogenic cysts. The cystic area in macroscopic images (arrowhead) and in panoramic radiographies (green rectangle) in odontogenic keratocyst (**A** and **B**, respectively), radicular cyst (**C** and **D**, respectively) and dentigerous cyst (**E** and **F**, respectively)

Macroscopic and radiological images of cysts were presented in Fig. 1. A huge cystic area was evident after removal of cysts in macroscopic images (Fig. 1A, C and E). Panoramic radiographies of keratocyst showed radiopaque borders with radiolucent content, resorbing the adjacent bone structures (Fig. 1B). In radiography of radicular cysts, radiopaque and prominent borders, radiolucent content, destructing the adjacent anatomical structures were evident (Fig. 1D). In radiological examinations of dentigerous cysts, smooth and radiopaque borders with radiolucent content were visible (Fig. 1F).

### Histopathological findings

**Fig. 2:**
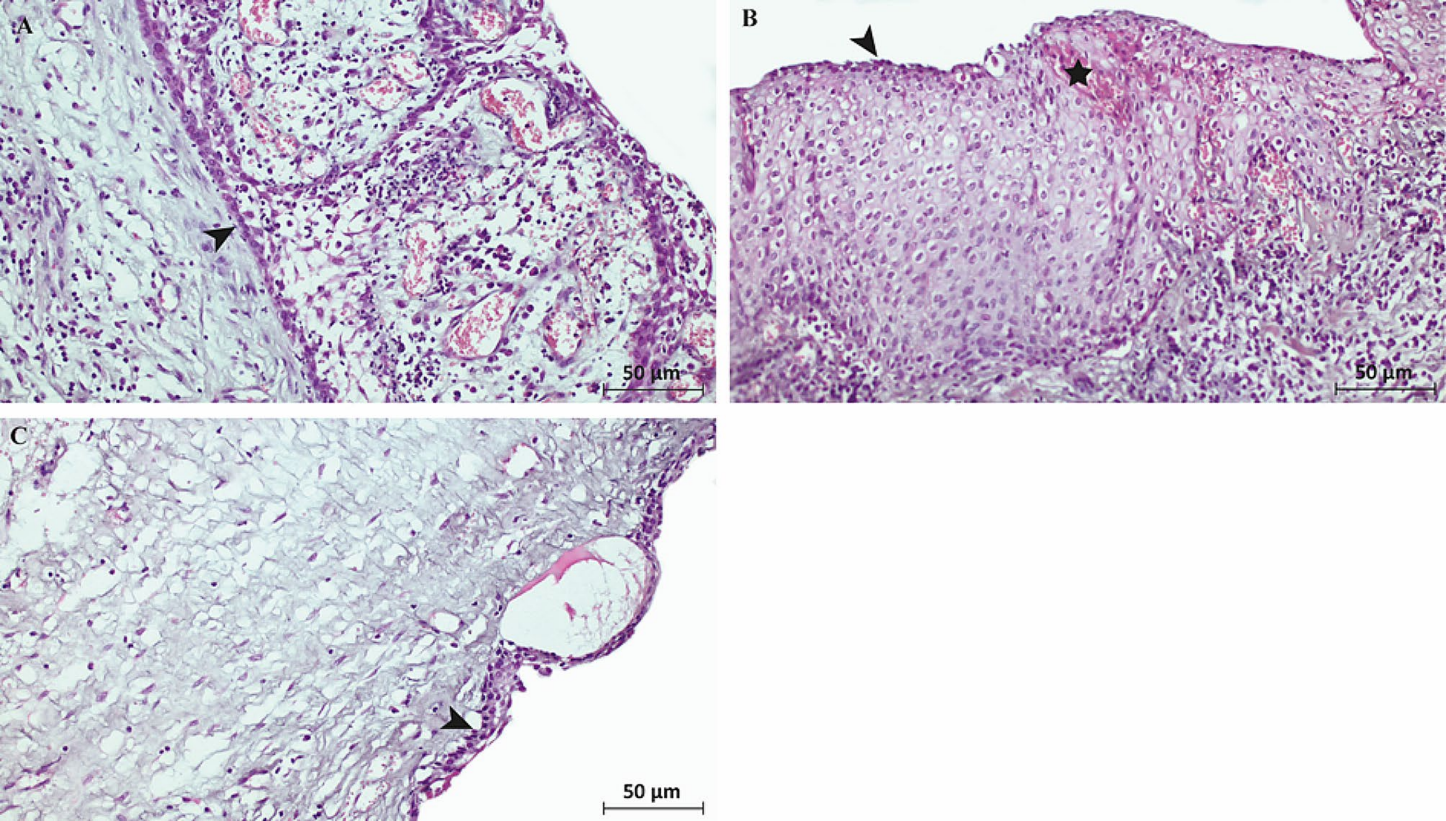
Sections of odontogenic cysts with Hematoxylin eosin staining. **(A) Keratocyst**: epithelial lining (arrowhead) without rete pegs; **(B) Radicular Cyst**: Epithelium with ciliated cells (arrowhead) and Rushton hyaline bodies (asterisk); **(C) Dentigerous Cyst**: Hyperplastic non-keratinized epithelium with rete ridges (arrowhead). Scale Bar: 50 μm, magnification: 20X

Histopathological images of odontogenic cysts were shown in Fig. 2. Sections of odontogenic keratocysts showed 6–8 layered epithelial cells without rete pegs, a cyst wall surrounded by fibrous tissue with inflammatory cells (Fig. 2A). In sections of radicular cysts, stratified squamous epithelium with different thicknesses and scattered ciliated cells and Rushton hyaline bodies were observed. The fibrous capsule and inflammatory cells were abundant (Fig. 2B). Sections of dentigerous cysts revealed that hyperplastic non-keratinized epithelium with fibrous tissue, rete ridges (pegs) and inflammatory cells (Fig. 2C).

**Fig. 3:**
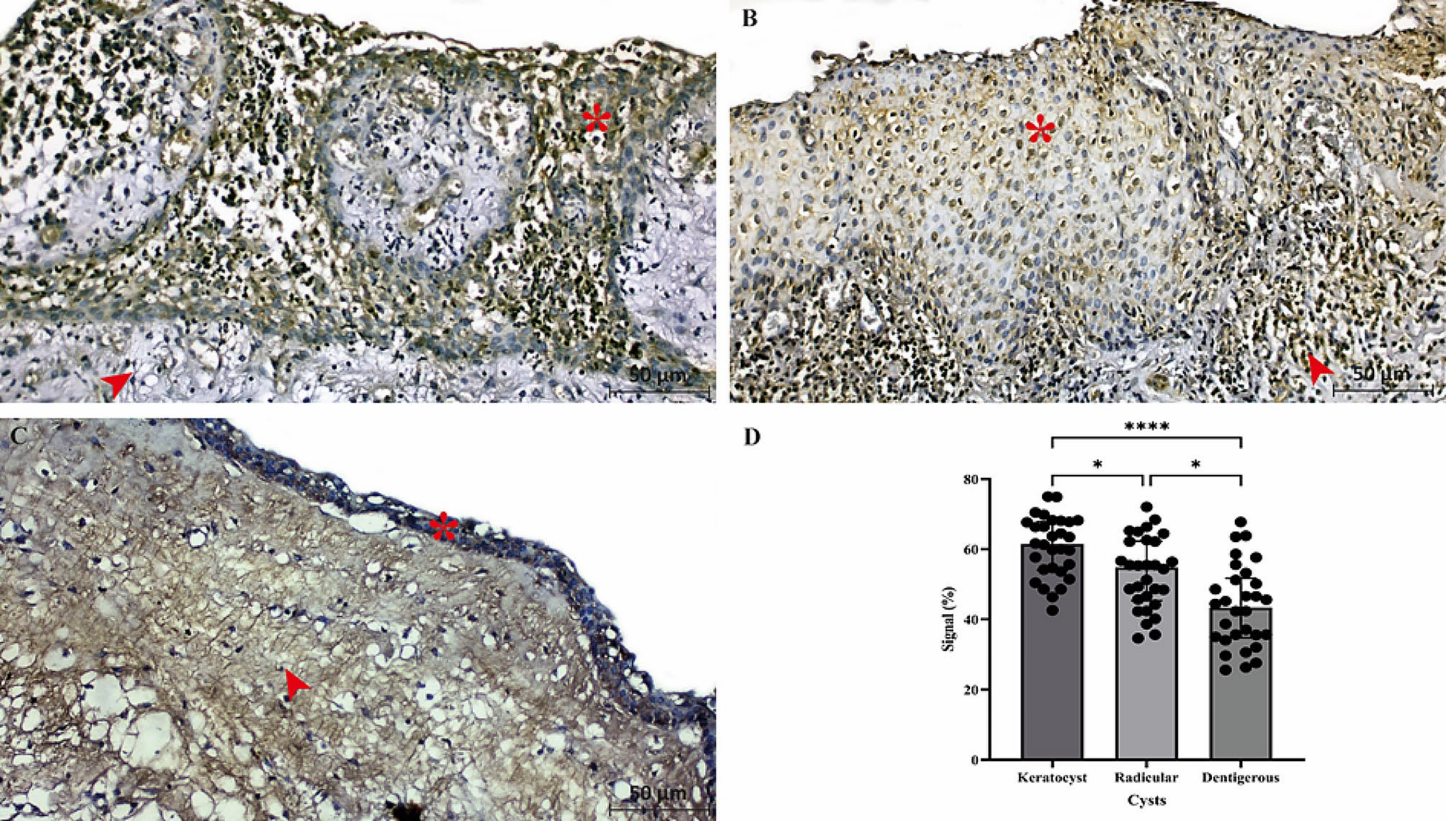
Sections of odontogenic cysts with CITED1 immunostaining. (**A**) Keratocyst, (**B**) Radicular cyst, (**C**) Dentigerous cyst. (**D**) Semi-quantitative CITED1 signal intensity in cyts. Arrow: Fibrous tissue cells, asterisk: epithelial layer. Scale Bar: 50 μm, magnification: 20X

**Fig. 4:**
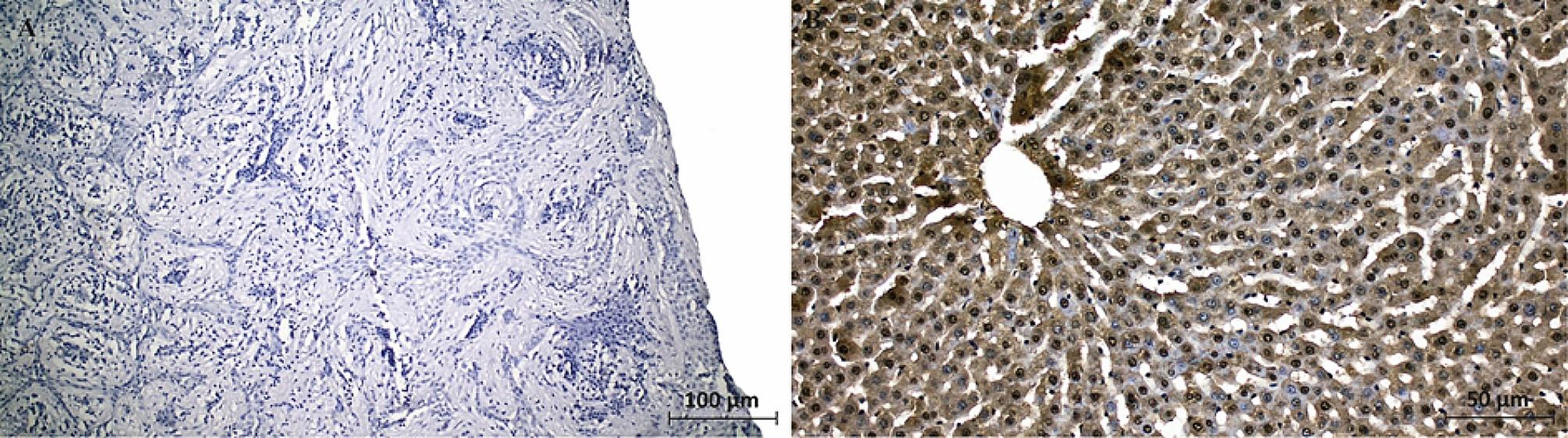
Negative (**A**) and positive (**B**) control for CITED1 antibody in different tissues

CITED1 immunoexpression was shown in Fig. 3. CITED1 expression was mainly observed in epithelial layers of cysts. Nuclear and cytoplasmic CITED1 expression was significantly higher in odontogenic keratocysts than in radicular and dentigerous cysts (Fig. 3A). Radicular cysts had significantly lower nuclear and cytoplasmic CITED1 expression than in odontogenic keratocysts (Fig. 3B). Dentigerous cysts significantly had the lowest nuclear and cytoplasmic CITED1 expression compared to odontogenic keratocysts and radicular cysts (Fig. 3C). Semi-quantitative analysis also confirmed significant CITED1 reactivity alteration between groups (Fig. 3D). CITED1 expression (signal) was the highest in odontogenic keratocyst, secondly in radicular cyst and the lowest in dentigerous cyst. Negative and positive control staining for CITED1 antibody were presented in Fig. 4. Dental cyst tissues were stained through all steps of the immunostaining procedure except the primary antibody was omitted for negative control. Liver tissue [24] was used to confirm that the staining procedure was working correctly as a positive control.

### Key interactors in CITED1 associated network and KEGG pathway analysis

**Fig. 5:**
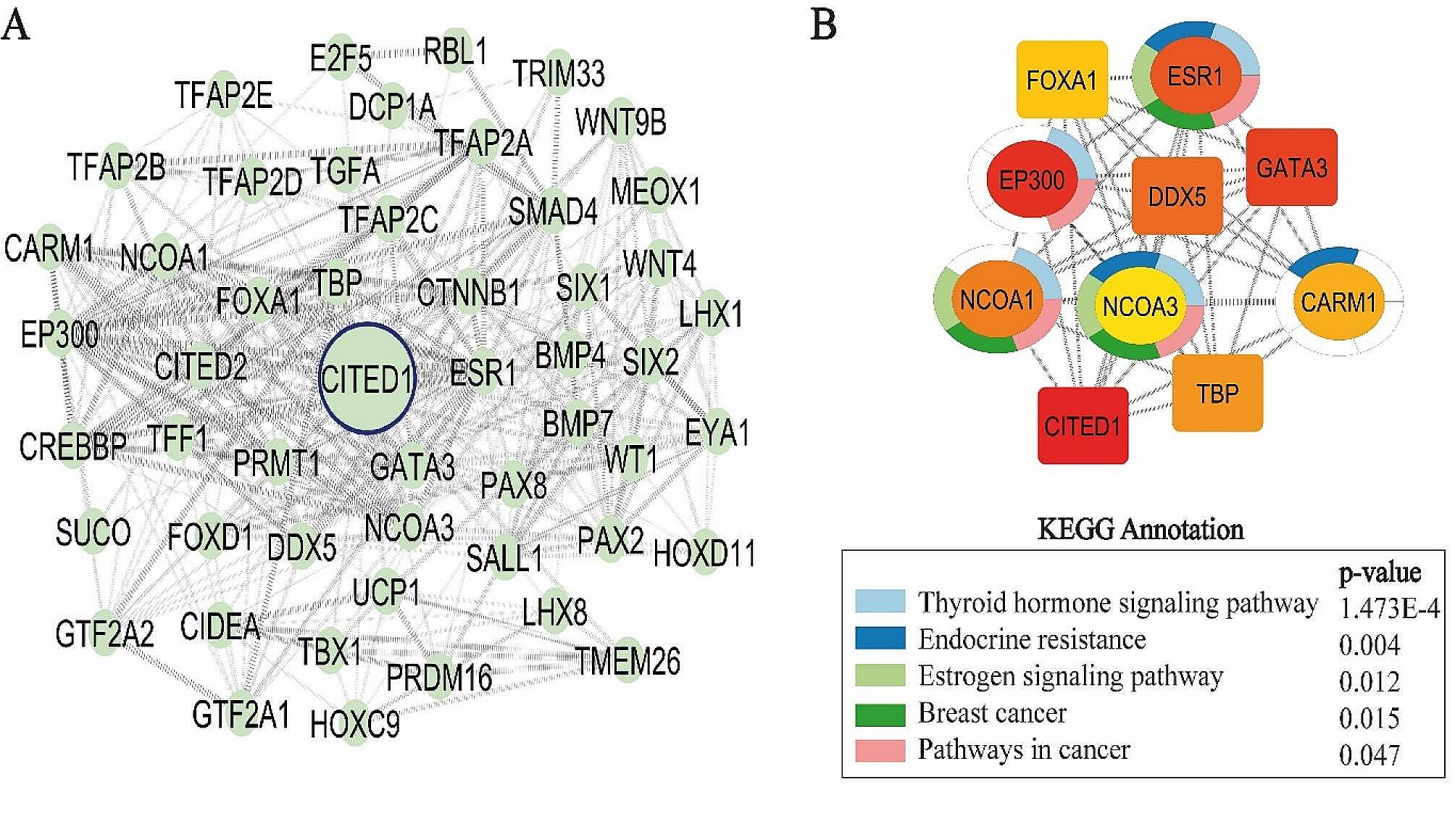
PPI network of CITED1 and KEGG pathway analysis. **A**. The protein interactors of CITED1 PPI network including 51 nodes and 354 edges. **B**. The PPI network of the ten hub genes and their KEGG pathway enrichment analysis. CytoHubba nodes are color-coded from red to yellow, representing the highest to lowest connection ranks according to the MCC algorithm. The significant five KEGG annotations are represented by colored ring graphs around the nodes (p<0.05)

After CITED1 PPI network was constructed and analyzed, we identified ten central interactors with the highest connectivity of this network. These proteins were as follows: NCOA1 (Nuclear receptor coactivator 1, Uniprot ID: ), CITED1(Uniprot ID: Q99966), DDX5 (DEAD-Box Helicase 5, Uniprot ID: P17844), KAT2B (Histone acetyltransferase KAT2B, Uniprot ID: Q92831), KAT5 (Histone acetyltransferase KAT5, Uniprot ID: Q92993), GATA3 (Trans-acting T-cell-specific transcription factor GATA-3, Uniprot ID: P23771), ESR1(Estrogen receptor, Uniprot ID: P03372), EP300 (Histone acetyltransferase p300, Uniprot ID: Q09472), TBP (TATA-box-binding protein, Uniprot ID: P20226), CARM1 (Histone-arginine methyltransferase CARM1, Uniprot ID: Q86 × 55). To identify signaling pathways correlated with these core proteins, we conducted KEGG analysis and revealed that they were significantly annotated with five pathways, “Thyroid hormone signaling pathway”, “Endocrine resistance”, “Estrogen signaling pathway”, “Breast cancer” and “Pathways in cancer” (*p* < 0.05) (Fig. 5).

## Discussion

Recent advances in imaging and screening technology provide a better understanding of the differentiation of odontogenic cysts [25]. However, still a histopathological confirmation is need for a definite diagnosis of odontogenic cysts. In this manner, molecular techniques can provide specific diagnosis for prognosis and treatment of cysts. This study investigated the keratocysts, radicular cysts, and dentigerous cysts with perspective of gross anatomy, radiology, histopathology, and immunohistochemistry. It is hard to distinguish odontogenic cysts from one another due to similar appearance of the stratified squamous epithelium and inflammatory reaction. Radiological and histopathological evaluation sometime may not be sufficient for odontogenic cysts [26]. In this study, the histopathological findings of odontogenic cysts and dentigerous cysts with inflammation were similar to radicular cysts causing the diagnosis harder. Differential histological diagnosis between developmental cysts with inflammatory infiltrate and inflammatory cysts is difficult since the morphological characteristics of the epithelial lining present changes due to inflammation, and “resemble” the epithelium of inflammatory odontogenic cysts [27]. Immunoexpression of proteins used as markers can alter their expression as a consequence of the inflammatory stimulus (example: inflammatory stimulus induces overexpression).Therefore, a useful method for diagnosis and treatment approaches for odontogenic cysts may be provided by immunohistochemical procedures.

CITED1 plays significant role in embryonic development and contributes to regulating the onset and progression of diverse tumors. Dahlgren et al. [28] found that high expression of CITED1 was associated in tamoxifen treated-patients with breast cancer. They proposed CITED1 could be a candidate for its potential diagnostic marker in breast cancer. Xu et al. [29] studied role of CITED1 in trophoblast lineages and recorded that CITED1 induced the trophoblast-like state in mouse embryonic stem cells and regulated trophoblast lineage specification through activating the BMP signaling pathway. A study examined the potential role of CITED1 in different malignant tumors of thyroid gland [30]. The authors revealed that most sensitive (%88 of cases) and specific (%92 of cases) marker was CITED1 for thyroid tumors. In a microarray analysis o9f melanoma study, Huang et al. [31] identified CITED1 as diagnostic or prognostic marker for melanoma.

Consistent with literature, CITED1 could be potentially a molecular marker for odontogenic cysts. In addition to embryogenesis, Role of CITED1 in inflammation is also under investigation. Subramani et al. [32] found that interferon gamma induced nuclear expression of CITED1 in macrophages adipose tissue and in response CITED1 accelerated the expression of interferon stimulated gene. In this study, CITED1 expression was found all odontogenic cysts, but was highest in keratocysts. Expression of CITED1 was higher in radicular cysts than in that of dentigerous cyst. Our findings showed there were differential expression of CITED1 among keratocysts, radicular cysts, and dentigerous cysts. We suggest that CITED1 could serve as a potential molecular marker for distinguishing between these cyst types. As a prognostic marker, the differential expression of CITED1 among these cyst types suggests that CITED1 immunohistochemistry could help in distinguishing between different types of odontogenic cysts. Higher expression levels of CITED1, especially in keratocysts, may indicate a more specific molecular signature associated with this type of cyst, facilitating more accurate and specific diagnoses. Clinically, CITED1 expression levels could complement traditional diagnostic methods and contribute to a more comprehensive assessment of odontogenic cysts. As a prognostic marker, the observation of elevated CITED1 expression in certain cyst types, such as keratocysts, which are known for their aggressive behavior and recurrence, suggests a potential prognostic value. Higher CITED1 expression levels, particularly in keratocysts, could serve as an indicator of more aggressive disease behavior, guiding clinicians in predicting the likelihood of recurrence or complications. This prognostic information could be valuable in developing specific treatment strategies and monitoring protocols for patients with odontogenic cysts.

Odontogenic cysts could be regarded as cysts with an inflammatory component. One factor contributing to the formation of these cysts is the infection of root canals resulting from caries or trauma-induced changes in the pulp. An etiological factor involves a low-virulence odontogenic infection of prolonged duration, affecting the side of the affected tooth root canal which often results from an accessory canal following pulp necrosis [33, 34]. These pathogen-related factors are adequate to initiate an inflammatory response and subsequent inflammation [35]. In order to elucidate the potential pathways through which CITED1 may exert its effects on inflammation, KEGG pathway analysis was conducted. Our pathway analysis demonstrated that the core molecular targets of CITED1 were significantly associated with thyroid hormone, endocrine resistance, estrogen, and cancer-related signaling pathways. Studies have shown that optimal thyroid hormone levels are necessary to ensure the maintenance of the immune response. In cancer and pathological processes, it has been revealed that thyroid hormones are involved in inflammation through NF-kB, p38MAPK, and JAK/STAT signaling pathways [36]. In addition to thyroid hormone, several other hormones, including estrogen, were found to play a significant role in the inflammatory response [37]. Although estrogens and other sex hormones are mainly considered to regulate the development of reproductive organs, it is now evident that these steroids have diverse effects on various systems. For instance, the estradiol form of estrogen has the ability to regulate proinflammatory signals. Additionally, certain estrogen receptors that mediate the effects of estrogens are frequently associated with anti-inflammatory characteristics [38]. On the other hand, carcinogenesis is also closely associated with inflammatory mechanisms. Various types of stimuli, such as carcinogenic microbes, environmental pollutants, and the breakdown of epithelial barriers associated with commensal microorganisms, might trigger inflammatory mechanisms within tumors [39]. Thus, the central interactors of CITED1 may also act as essential modulators of inflammation.

Given the potential association between CITED1 expression and inflammation, as suggested by previous studies and our conducted KEGG analysis of core molecular targets of CITED1, further investigation into the inflammatory mechanisms underlying odontogenic cyst formation and progression could provide additional insights into the role of CITED1 in cyst pathophysiology. This understanding may lead to the development of anti-inflammatory therapies targeting CITED1-mediated pathways.

Overall, incorporating the expression patterns of CITED1 into clinical practice may enhance diagnostic accuracy, guide treatment decisions, and improve prognostic assessments for patients with odontogenic cysts. However, further research is needed to validate these findings and explore the therapeutic implications of targeting CITED1 in odontogenic cyst management.

### Limitations

The study utilized a relatively small sample size of 50 cases for each type of the odontogenic cysts. This limited sample size might not fully represent the variability within each cyst type and could affect the generalizability of the findings. The study was conducted in a single center, which might introduce bias related to patient demographics, clinical practices, and specimen handling procedures. The immunostaining technique used to detect CITED1 expression might have limitations in terms of sensitivity and specificity. The study adopted a cross-sectional design, however longitudinal studies are needed to explore changes in CITED1 expression over time and its association with disease progression. Although PPI network provided predicted CITED1 interactors through bioinformatic analyses, further research is needed to elucidate the mechanistic pathways through which CITEDs may contribute to cyst development and progression.

## Conclusions

It is evident that CITED1 expression was detected in all types of odontogenic cysts studied. However, the expression levels varied among the different cyst types. Specifically, the highest expression of CITED1 was observed in keratocysts, indicating a potentially significant role of CITED1 in the pathogenesis of these cysts. Furthermore, the expression of CITED1 was found to be higher in radicular cysts compared to dentigerous cysts, suggesting differential regulatory mechanisms among various types of odontogenic cysts. These findings highlight the potential relevance of CITED1 in the development and progression of odontogenic cysts, with further implications for understanding their pathophysiology and potential therapeutic targets.

## Data Availability

All data generated or analysed during this study are included in this published article.
